# Reduced myocardial reserve in cirrhotic patients: an evaluation by dobutamine stress speckle tracking and tissue Doppler imaging (TDI) echocardiography

**DOI:** 10.15171/jcvtr.2019.22

**Published:** 2019-06-22

**Authors:** Mahmood Zamirian, Forough Afsharizadeh, Alireza Moaref, Firoozeh Abtahi, Fatemeh Amirmoezi, Armin Attar

**Affiliations:** ^1^Department of Cardiovascular Medicine, Shiraz University of Medical Sciences, Shiraz, Iran; ^2^Cardiovascular Research Center, Shiraz University of Medical Sciences, Shiraz, Iran; ^3^Students’ Research Committee, Shiraz University of Medical Sciences, Shiraz, Iran

**Keywords:** Cirrhosis, Global Strain, Myocardial Reserve, Speckle Tracking, Echocardiography

## Abstract

***Introduction:*** Despite the normal systolic function at rest, cirrhotic patients often suffer from volume overload and symptoms of heart failure as they face stressful situations. This study investigated the myocardial reserve in cirrhotic patients at resting condition and peak stress by dobutamine speckle tracking echocardiography (STE) and tissue Doppler imaging (TDI).

***Methods:*** Twenty cirrhotic patients and 10 normal individuals aged 30-50 were selected randomly. For all of the participants, complete echocardiographic study of 2D, STE and TDI was done at rest and peak stress status with dobutamine. The following parameters were assessed: ejection fraction (EF), global longitudinal LV strain (GLS), strain rate in the septal basal segment and lateral wall and E’ in the septal basal segment by color-coded method.

***Results:*** At baseline, EF was higher than 55% in both groups. GLS was higher (-22.6±2.4%) in the case group than the control group (-19.2±1.9%) at resting condition. After stress, it showed a greater increase (-22.5±1.7%) in the controls compared to cirrhotic patients (-22.6±3.3%; mean difference = 2.6 ± 2.03, *P *= 0.02). In cirrhotic patients, the average strain rate in the basal septal segment decreased after stress (-1.2 ± 0.3/s to-1.1 ± 0.3/s), but it increased in the control group (-1.1 ± 0.2/s to -1.8 ± 0.2/s).

***Conclusion:*** Despite the presence of normal resting systolic function in cirrhotic patients, there was insufficient increase or even a decrease in myocardial function with stress; this may indicate the absence of sufficient myocardial reserve in cirrhotic patients. These findings would help to explain the reason for occurrence of heart failure or hemodynamic changes in cirrhotic patients.

## Introduction

Cirrhotic cardiomyopathy is a form of cardiac dysfunction in cirrhotic patients in the absence of other known cardiovascular diseases. Cirrhosis may have adverse effects on the main organs of the body. In these patients, a series of hemodynamic changes occurs in the form of increase in cardiac output and heart rate, reduction in systemic vascular resistance, reduced or normal arterial pressure, and visceral artery vasodilatation which may lead to hyper-dynamic syndromes at resting condition.^1,2^ In these patients, despite the increase in the cardiac output and ventricular contraction at resting condition (due to the hyper-dynamic flow of the blood), in physiological, pharmacologic or pathologic stressful situations such as liver transplant and trans-jugular interahepatic portosystemic shunt (TIPS) implantation, the symptoms of heart failure may appear. This condition is called latent heart failure or cirrhotic cardiomyopathy.

After liver transplantation, heart failure symptoms may also appear due to the rapid increase in the venous return, volume overload, and increase in the strain of the heart. Consequently, stroke volume may reduce because of impaired myocardial contractility or reduced preload due to bleeding during surgery or fluid retention in the third space and could lead to the death of the patients after liver transplantation^3-5^; however, most of the time, these events are subclinical.^6,7^

Diastolic dysfunction which is more common than systolic dysfunction in cirrhotic patients occurs due to increased stiffness of the myocardium followed by hypertrophy or edema which could be due to sodium retention, fluid retention, and activation of a series of neuro-hormonal systems as well as the increased effect of fibrotic aldosterone by sodium.^3,8^

Although considerable advances are made regarding the pathophysiology of cirrhotic cardiomyopathy, clinical signs and its side effects, there is no certain diagnostic test for identifying the patients at risk of heart failure after liver transplant, so that it could help to act upon early diagnosis and treatment and improved prognosis of the patients. In this study, we aimed to compare the cardiac function of the cirrhotic patients who were candidate of liver transplantation with the new and advanced methods of echocardiography and compare the results with the that of the normal people to find clues for explanation of cirrhotic cardiomyopathy.

The most common method for qualitative evaluation of the LV is ejection fraction (EF); however, evaluation with the traditional two-dimensional (2D) echocardiography does not have sufficient sensitivity to diagnose the myocardial dysfunctions in the early stages.^6,9^ One of the new modalities in the evaluation of myocardial performance is to measure the values of myocardial strain or regional deformation in the myocardium which is an expression in echocardiography to describe the length of certain parts of the myocardium. A new method for measuring the strain is speckle tracking echocardiography or STE.^10,11^ Therefore, we decided to measure the values of strain rate, global LV strain (as an indicator of the systolic function which is relatively independent of the load) at resting condition and in stressful conditions to have a more precise evaluation of cardiac function in cirrhotic patients.

## Materials and Methods

### Patients

This cross-sectional study was carried out from the 21st of April to 22nd of December in 2013 in Shiraz University teaching hospital in Iran. Cirrhotic patients who were in the waiting list for liver transplantation, were referred for heart consultations, and were eligible for the study were selected by simple or convenience method. The age range of these patients was 30-50 years. The cause of cirrhosis was diverse in this study and written consent forms were obtained from all participants. Also, normal individuals were selected as the control group with an age range of 30-50 who were volunteers to be considered in the study and were eligible. Exclusion criteria included history of coronary artery disease, clinical and preclinical symptoms or evidence of coronary artery disease, underlying arrhythmias or history of arrhythmia, severe valvular disease, congenital heart diseases, EF below 55%, and diabetes. This study conforms with the declaration of Helsinki about working with human subjects and the local Ethics Committee has approved the study protocol and patients or their relatives gave informed consent.

### Echocardiographic studies

For all the patients and the control group, routine echocardiography was performed using Vivid E9 model device and parasternal, apical and sub-costal images were taken for all the patients while lying on their left and the dimensions of the LV were measured in systolic and diastolic phases. Then, the images were stored in three views of apical 4-ch, apical 2-ch and apical long axis with frame rates of about 50-60 and global systolic longitudinal LV strain of the LV was measured and demonstrated as bull’s eye diagram and E'^1^ parameter was measured in the septal basal segment for the investigation of diastolic function using color-coded method. Also, the strain rate in the septal basal segment and lateral wall was measured using tissue Doppler imaging (TDI) and the data were stored.

Then, the patients were studied under stress condition with dobutamine according to the planned protocol and longitudinal LV strain of the left ventricle was again measured globally in the form of bull’s eye; the strain rate was also measured and stored in the septal basal segment and the lateral wall.

Also, to study the diastolic function, E’ was studied using color-coded method and the E’s with values of ≥6 m/sec were compared (25% of the values by pulse wave TDI). Dobutamine prescription protocol for investigating the myocardial reserve based on the guideline of the American society of echocardiography was carried out as follows^12^:

First, dobutamine infusion starts with 5 µg/kg/min and the dosage of dobutamine was increased by 3-5 µg/kg/min every 3 minutes to reach the maximum dosage of 15-20 µg/kg/min. With this dosage of dobutamine, the LV contractility was enhanced, but the heart rate of the studied individuals did not change significantly and the parameters were recorded at the peak stress and the individuals were monitored for their ECG and blood pressure.

### Statistical analysis

For comparing the groups of patients and control in terms of global strain, strain rate and E’ values were done using Mann-Whitney test. Fisher’s exact test was used for the comparison of gender distribution and diastolic function. *P* value < 0.05 was considered statistically significant. All data were analyzed using the statistical Package for Social Sciences, version 17.0 (SPSS Inc., Chicago, IL, USA).

## Results

A total of 30 people (20 patients with liver cirrhosis and 10 normal people as the control group) were examined in this study; their distribution in terms of gender and age is illustrated in Table 1. There was no statistically meaningful discrepancy in gender and age (Table 1). Neither the patients nor the control group faced hypotension, hypertension, significant arrhythmia, angina, significant changes in the ECG, or new well motioned abnormality in at least two segments.

**Table 1:** Baseline demographic data

	**Cirrhosis**	**Control**	***P*** ** value**
Man (%)	10 (50%)	8 (80%)	0.23
Woman (%)	10 (50%)	2 (20%)	0.23
Age	42.2±4.7	41.6±4.7	0.58
Blood urea nitrogen	11.6±5.6	16.3±9.8	0.23
Creatinine	0.65±0.2	0.75±0.3	0.12
Aspartate aminotransferase	184.3±36.8	23.6±11.2	<0.001
Alanine aminotransferase	168.4±43.5	25.4±10.6	<0.001
Alkaline phosphatase	1174.3±250.4	65.6±21.4	<0.001
Albumin	4.07±1.2	4.5±1.4	0.21
Prothrombin time	18.4±2.6	11.2±1.3	0.02

In each group (liver cirrhotic patients and control group), the global longitudinal LV strain was measured at resting condition. The mean global strain for the cirrhotic patients was higher compared to the control group at resting condition which was statistically significant. However, the difference of global strain between the two groups following stress (infusion dobutamine peak according to the protocol) was not significant (Table 2).

**Table 2:** Comparison of the mean and standard deviation of different echocardiographic parameters in cirrhotic patients and control groups

**Parameter**	**Group**	**Mean ± SD**				**Differences**	***P*** ** value**
		**At rest**	***P*** ** value**	**At peak stress**	***P*** ** value**		
Global longitudinal strain (%)	Cirrhosis	-22.6±2.4	0.001^*^	-22.6±3.3	0.071	0.7±2.1	<0.001^*^
	Control	-19.2±1.9		-22.5±1.7		3.3±1.2	
The strain rate (s-1) of septal basal segment	Cirrhosis	-1.2±0.3	0.183	-1.1± 0.3	<0.001^*^	-0.1±0.4	<0.001^*^
	Control	-1.1±0.2		-1.8±0.2		0.6±0.2	
The strain rate (s-1) of lateral segment	Cirrhosis	-1.4±0.5	0.687	-1.5±0.7	-0.055	0.1±0.6	0.024^*^
	Control	-1.4±0.2		-1.8±0.2		0.4±0.2	
E' (cm/s) of septal basal segment	Cirrhosis	7.3±1.7	0.286	7.4±1.8	0.028^*^	0.1±0.3	0.005^*^
	Control	7.7±1.3		9±1.7		1.3±0.6	

The global strain variations showed less increase at peak stress in the patients group compared to the control group and it even decreased in some cases which was statistically significant. The strain rate with TDI method in the lateral and septal basal segment at resting condition showed no significant variation between the two groups. The mean strain rates at peak stress between cirrhotic and control groups were significantly different for the lateral and septal basal segment and the strain rate was higher in the control group (Table 2).

The strain rate at peak stress when compared with resting condition decreased in cirrhotic patients in the septal basal segment and it increased in the control group (*P* value <0.001). Also, the strain rate at peak stress in the lateral wall increased in each group compared to the values at resting condition, but the increase was higher in the control group (Table 2).

No statistically significant discrepancy existed in E’ (early diastolic velocity) between groups at resting condition in the septal basal segment using color-coded imaging. Even so, this parameter at peak stress was statistically significant and E’ was higher in the control group (Table 2). Comparison of the mean E’ at resting condition and peak stress in both groups showed that E’ in the control group had increased more following stress and the difference was statistically significant.

Four patients among cirrhotic patients (20%) had diastolic dysfunction and 1 person in the control group (10%) had diastolic dysfunction which showed no statistically significant discrepancy. Also in peak stress status, 6 persons among cirrhotic patients (30%) had diastolic dysfunction and all the participants in the control group had normal diastolic function and the difference was not statistically significant (Table 3). Therefore, the number of cirrhotic patients with diastolic dysfunction following stress increased, but all the participants in the control group had normal diastolic function at peak stress.

**Table 3:** Study of the diastolic dysfunction at resting condition and peak stress in the two groups (based on the study of E’)

	**Groups**	**Diastolic function**		**P value**
		Normal	Abnormal	
Rest condition	Cirrhosis	16 (80%)	4 (20%)	0.64
	Control	9 (90%)	1 (10%)	
Peak stress	Cirrhosis	14 (70%)	6 (30%)	0.07
	Control	10 (100%)	0 (0%)	

All the patients has normal systolic pulmonary artery pressure before and after stress.

## Discussion

Our findings suggest that despite the normal or even increased EF (>55%) and global strain at resting condition in most of the cirrhotic patients, mean global longitudinal LV strain did not increase appropriately following stress stimulation (dobutamine infusion according to the protocol) and it even decreased. Moreover, diastolic function in the control group was normal at peak stress; however, 30% of the cirrhotic patients developed diastolic dysfunction following stress. In other words, both systolic and diastolic dysfunction appeared in the cirrhosis group following stress.

According to the obtained results in the current study, LV systolic function (EF) in all the studied cirrhotic patients and control group at resting condition was normal or higher than normal (EF > 55%). Our results were consistent with that of a recent study which assessed 44 cirrhotic patients by conventional echocardiography and TDI. In that study, despite a normal EF at resting condition, the strain rate and peak systolic tissue velocity (systolic function criteria) were decreased.^6^ Therefore, two-dimensional echocardiography is not an appropriate modality for detection of early stage of cardiomyopathy in cirrhosis, as our findings indicated.^6,13^ Consequently, using newer and more precise techniques are needed to evaluate cardiac function in cirrhotic patients.

Two-dimensional STI was the modality that Xu et al used to evaluate the cardiac function of cirrhotic patients. They concluded that 2D-STI can early and accurately evaluate the systolic function of liver cirrhosis compared to 2D echocardiography.^9^ Our results implied that according to tissue-Doppler and speckle-tracking analysis, global systolic longitudinal strain in patients with liver cirrhosis was higher than that in healthy ones at resting conditions. In contrast with our results, Sampaio et al implied that at resting condition, global systolic longitudinal strain in patients with liver cirrhosis was lower than that in healthy ones in spite of normal EF values.^14^

The first presentation of cardiac impairment is reduced LV longitudinal in several other conditions, such as cardiomyopathy due to anthracycline consumption.^3,14^ Presence of abnormal strain rates despite normal EFs may be related to the tissue structure of the myocardium. Subendocardial fibers are at the farthest point to vascular supply; therefore, longitudinal left ventricular fibers are more susceptible to damage than radial middle fibers.^14^ The 2005 World Congress of Gastroenterology proposed a resting EF <55% to define systolic dysfunction in patients with cirrhosis.^15^ The use of EF in evaluating the systolic function has several limitations because it is not an index of contractility and depends on the volume loading, heart rate and valvular function.^16^ Therefore, a ‘normal’ EF may not reflect normal contractile function and this may be particularly true in cirrhosis. Our finding suggests that EF may be an insensitive (albeit specific) and probably late marker of systolic dysfunction in cirrhosis, identifying only patients with more severe myocardial dysfunction.

Inducing stress to evaluate the cardiac function may be the most precise way to reveal decreased myocardial reserve. To the best of our knowledge, our study is the first to use stress echo and speckle tracking to evaluate myocardial reserve in cirrhotic patients. Here, we have shown that despite higher values at resting condition, global longitudinal strain rate is not increased or even decreased at peak stress in cirrhotic patients as compared to controls. In the same line with our findings, pharmacological stress tests using dobutamine was used in a study with cardiovascular magnetic resonance imaging in cirrhotic patients. It was shown that during low and intermediate dose of dobutamine, the global longitudinal strain was impaired.^17^ Considering the lower costs, higher availability, and easier preformation, dobutamine stress STE is a more logical way to reveal latent myocardial dysfunction in cirrhotic patients.

In our study, we also noticed that with stress 10% of cirrhotic patients developed diastolic dysfunction. Kim et al evaluated the LV systolic and diastolic functions by two-dimensional and Doppler echocardiography at rest and during peak dobutamine infusion. In agreement with our findings, their results showed that 25% of cirrhotic patients had diastolic dysfunction after dobutamine stimulation that was compatible with our findings.^18-20^

Our study had some limitations. As a case control study, we could not exclude the effect of various pathological conditions that had led to liver failure. However, one should notice that these effects are part of the cirrhosis and the eventual result irrespective of the cause of liver dysfunction. Also role of failure to achieve to total heart reserve as a main cause of poor myocardial reserve and inappropriate rise in Strain cannot be excluded.

It can be concluded that despite the presence of normal resting cardiac function in cirrhotic patients, there is insufficient increase or even a decrease in myocardial function with stress which may indicate absence of sufficient myocardial reserve in cirrhotic patients. This may help explain why cirrhotic patients may show signs of heart failure or hemodynamic disorders following stress (e.g. after liver transplant surgery, TIPS implantation and gastrointestinal bleeding etc.). In fact, these patients suffer from subclinical and latent LV disorders. Moreover, the conventional two-dimensional echocardiography is not a sensitive modality for evaluation of systolic dysfunction and myocardial reserve and using stress STE may be the logical way.

## Ethical approval

The study was approved by medical ethics committee of the university (ethics No. 4955).

## Competing interests

All authors declare no competing financial interests exist.

## Acknowledgment

This project has been done by a grant No. 4955 from Vice-chancellor of Shiraz University of Medical Sciences. The authors are grateful to Center for Development of Clinical Research of Nemazee Hospital and Dr. Nasrin Shokrpour for editorial assistance.
